# Family Socioeconomic Status and General Self‐Efficacy in Nursing Students: A Moderated Mediation Model of Family Relationship Quality and Institutional Type

**DOI:** 10.1155/jonm/9942454

**Published:** 2026-03-31

**Authors:** Anshi Wang, Lijun Zhu, Xuting Dong, Liying Wen, Weiwei Chang, Yu Zhu, Yuelong Jin, Jingjing Wan

**Affiliations:** ^1^ School of Public Health, Wannan Medical College, Wuhu, China, wnmc.edu.cn; ^2^ School of Nursing, Anhui College of Traditional Chinese Medicine, Wuhu, China

**Keywords:** family relationship quality, family socioeconomic status, general self-efficacy, institution type, moderated mediation, nursing students

## Abstract

**Background:**

Nursing students face significant psychological challenges, with general self‐efficacy (GSE) serving as a critical protective factor. Although family socioeconomic status (SES) and family relationship quality (FRQ) have been linked to GSE, their underlying pathways and the moderating role of institution type (vocational vs. undergraduate colleges) remain unclear. This study aimed to examine the association between SES and GSE among nursing students, focusing on FRQ as a mediator and institution type as a moderator.

**Methods:**

A cross‐sectional survey of 1461 nursing students from two Chinese medical institutions was conducted. SES was derived from five socioeconomic indicators using principal component analysis. FRQ was constructed as a latent variable from three observed items using confirmatory factor analysis. GSE was measured using the General Self‐Efficacy Scale. Mediation and moderated mediation analyses were performed using PROCESS macros.

**Results:**

SES was positively associated with GSE (*B* = 0.313, *p* = 0.004). FRQ partially mediated the relationship between SES and GSE in the total sample (*β* = 0.060, 95% CI [0.009, 0.115]). However, this mediation was only significant among undergraduates (*β* = 0.118, 95% CI [0.043, 0.201]) and not among vocational students (*β* = −0.005, 95% CI [−0.060, 0.051]). Institution type moderated both the direct effect of SES on GSE and the indirect effect via FRQ, with stronger associations among undergraduate students. Specifically, simple slope test showed that SES was associated with GSE among undergraduates (*β* = 0.455, 95% CI [0.191, 0.720]) but not among vocational students (*β* = 0.021, 95% CI [−0.324, 0.367]).

**Conclusions:**

Family SES was positively associated with nursing students’ GSE directly and indirectly via FRQ, and these associations were stronger among undergraduate than among vocational students. These findings suggest that considering both family resources and educational context may be useful when designing targeted interventions to foster GSE among nursing students.

## 1. Introduction

As an essential pipeline for the future healthcare workforce, nursing students are increasingly recognized as a population of global concern due to their vulnerability to psychological distress. Due to the distinctive nature of the profession, nursing students are exposed to multiple stressors, including high‐intensity academic pressure, highly stressful clinical practice environments, ethical dilemmas, moral challenges, substantial emotional demands, and uncertainty about future career prospects [1–3]. Consequently, the prevalence of mental health issues among this group is significantly higher than that of the general university student population [4–6]. Given these circumstances, identifying protective factors that effectively maintain and enhance the psychological health of nursing students has emerged as an urgent priority.

General self‐efficacy (GSE) is defined as a stable, cross‐domain belief in one’s competence to mobilize motivation, cognitive resources, and courses of action required to exercise control over task demands and environmental challenges [7]. Research has demonstrated that GSE is closely associated with stress coping and psychological resilience, playing a significant role in mitigating mental health problems [8–10]. Individuals with high levels of GSE generally approach challenges with optimism and perseverance and exhibit confidence in their ability to manage difficulties. Therefore, investigating the factors influencing GSE holds considerable theoretical and practical significance.

## 2. Background

To address the urgent need to identify protective factors for nursing students’ psychological health, it is essential to clarify the underlying factors associated with GSE, a key buffer against their professional stressors. Existing studies have confirmed that both family socioeconomic status (SES) and family relationship quality (FRQ) are associated with GSE [11, 12]. SES is a comprehensive measure of a family’s economic, educational, and occupational background and is often closely linked to resource acquisition and developmental opportunities [13]. FRQ, on the other hand, reflects the quality of an individual’s interactions with their parents and the harmony of the parental relationship, representing a core aspect of emotional support within the family environment [14]. As two complementary constructs, SES and FRQ capture structural/material and relational dimensions of the family environment, respectively. SES emphasizes access to material and social resources (e.g., educational investment and economic capital), while FRQ focuses on emotional support mechanisms (e.g., parent–child interaction and family cohesion). This two‐dimensional framework facilitates a more comprehensive understanding of how family factors are associated with GSE through multiple pathways and offers a theoretical basis for developing differentiated intervention strategies. In this study, SES was operationalized using residence (urban/town/rural) along with parental education and occupation, reflecting structural/material family resources. FRQ captures relational functioning within the family system (e.g., parent–child relationship quality and parental relationship quality) and is related to, but distinct from, perceived social support.

Students from high SES background typically have access to more abundant educational resources (e.g., high‐quality learning environments and extracurricular tutoring) and social capital (e.g., parental social networks and professional backgrounds). These resources may be associated with an individual’s high GSE and strengthen their confidence in facing challenges [15, 16]. This mechanism aligns closely with the Conservation of Resources (COR) theory. COR theory defines valued resources broadly, spanning entities, conditions, traits, and energies (e.g., objects, employment, skills, and money). The theory further holds that the pursuit of these resources through their acquisition, retention, and optimization is instrumental for developing the adaptability and capacity needed to navigate life’s challenges [17]. The superior educational and social resources accessible to students from high SES backgrounds functions as a vital asset reservoir. This reservoir not only facilitates resource accumulation and preservation but also fundamentally bolsters their perceived capability and assurance when navigating difficulties. This perspective is consistent with the central tenet of COR theory, which posits that resources facilitate environmental adaptation, promote change, and mitigate stress [17].

SES may also be linked to GSE through FRQ. Families with high SES (particularly higher household income) often experience less economic pressure, which has been linked to lower parental psychological distress (e.g., anxiety and depression) and better marital satisfaction [18]. Marital quality, a core indicator of FRQ, reflects how high SES fosters positive family dynamics [19]. As a key component of SES, abundant social capital strengthens family cohesion and emotional support, which is highly consistent with the concept of FRQ [20]. High SES families may be better equipped to provide structured role support (e.g., clear parenting responsibilities and resource allocation) and high family integration (e.g., emotional sharing and joint participation in activities), thereby creating a supportive and low‐conflict family environment [21]. These mechanisms collectively reduce the risks commonly associated with adverse home environments in low SES households, such as household chaos and poor marital quality [22], thereby improving overall FRQ. Furthermore, better FRQ positively influences GSE through multiple pathways. In a family environment characterized by low parental conflict and high relational satisfaction, parents are more likely to exhibit patient parenting behaviors and provide stable emotional support [18]. High levels of family cohesion, emotional sharing, and shared activities offer individuals consistent and reliable emotional support [20, 21]. Meanwhile, the improved frequency and quality of parent–child interactions facilitated by high SES further strengthen the family’s emotional support function [23]. Such a supportive, well‐structured, and emotionally responsive family environment provides individuals (especially children) with ample successful experiences and sources of confidence in tackling challenges. This effectively promotes positive self‐perceptions and evaluations of one’s capabilities, thereby contributing to the development and enhancement of GSE [18, 21]. In summary, SES is not only directly associated with resource acquisition but also helps establish a solid foundation for the development of GSE by shaping high FRQ as a crucial psychosocial support mechanism.

Institutions of higher education, characterized by distinct training models, differ in teaching resources, academic environments, educational approaches, and student demographics [24, 25]. These variations may further moderate the extent to which SES and FRQ are linked to GSE among nursing students. The pedagogical model in vocational colleges often prioritizes experiential learning and curriculum customization aligned with students’ baseline competencies. This approach fosters self‐efficacy through successful problem resolution in clinical scenarios, thereby cultivating a tangible sense of achievement [7]. This approach may partially mitigate the disadvantages in ability development associated with lower SES. In contrast, undergraduate nursing students face dual pressures from academic and clinical demands, including test anxiety and heavy academic workloads, making GSE a crucial psychological resource for coping with stress [9]. Students from high SES families in undergraduate programs, being less concerned about resource scarcity, are often more focused on competency development; encouragement and feedback from high FRQ families can further strengthen their self‐efficacy [11]. Accordingly, family‐derived resources and supportive family relationships may play a more salient role in sustaining students’ GSE.

However, several research gaps remain: (1) The mediating mechanism has not been verified: whether FRQ mediates the relationship between SES and GSE has not been examined among nursing students. (2) The moderating effect is unclear: whether the institution type moderates the direct path, the mediating path, or the entire chained mechanism remains uncertain. Guided by COR theory, we integrate structural and relational family resources and test a moderated mediation mechanism in which educational context (institution type) conditions the direct and indirect associations between family resources (SES and FRQ) and GSE. Accordingly, we proposed the following hypotheses: Hypothesis 1: SES significantly and positively predicts GSE among nursing students (direct effect); Hypothesis 2: FRQ mediates the relationship between SES and GSE (indirect effect); Hypothesis 3: Institution type moderates the direct effect of SES on GSE, with a stronger effect among undergraduate students; Hypothesis 4: Institution type moderates the mediating path, with a stronger indirect effect among undergraduate students. By clarifying these relationships, this study aims to provide a theoretical foundation and practical intervention strategies for enhancing GSE among nursing students.

## 3. Methods

### 3.1. Study Designs and Participants

This cross‐sectional study assessed the mental health status of nursing students between March and June 2024. Participants were recruited from nursing programs at two Chinese medical higher education institutions: one offering a 3‐year vocational diploma and the other a 4‐year undergraduate bachelor’s degree. The two institutions were purposively selected because they represent typical vocational and undergraduate nursing education pathways within the same geographic region, which helped reduce regional and policy heterogeneity while enabling an initial comparison of institution type differences. Nevertheless, we acknowledge that sampling only one school per institution type may not capture the full heterogeneity within each educational track.

A multistage sampling strategy was implemented, and the minimum sample size was calculated using the standard formula for categorical outcomes in cross‐sectional studies: *n* = *t* ^2^ *p*(1 − *p*)/*d* ^2^ (*t* = 1.96, *p* = prevalence of the outcome of interest, *d* = margin of error, defined as 10% of *p*). Based on preliminary survey data, the estimated prevalence rates were anxiety: 35%, depression: 30%, and stress: 25%. To ensure adequate statistical power across all measured outcomes, the most conservative estimate (*p* = 25%) was applied. Additionally, accounting for an anticipated 20% attrition rate, the adjusted minimum sample size was 1500. The study successfully recruited 1550 participants from 50 classes, achieving 100% questionnaire retrieval. Following rigorous quality control, 89 invalid responses (40 predominantly blank, 49 exhibiting systematic response patterns) were excluded, resulting in 1461 valid questionnaires (94.26% valid response rate) for analysis.

The study protocol was approved by the Ethics Committee of the School of Public Health, Wannan Medical College (Approval No. LL‐WK2024ZQNZ16) and complied with the Declaration of Helsinki. All participants provided written informed consent.

### 3.2. Instruments

Fourteen general demographic characteristics were collected via questionnaire: age, grade, gender (male/female), only‐child status (yes/no), residence (rural/town/urban), relationship with father (1 = Very Poor, 5 = Very Good), relationship with mother (1 = Very Poor, 5 = Very Good), parental relationship (1 = Very Poor, 5 = Very Good), father’s education (primary school or below/junior high school/senior high school or technical secondary school/associate degree or higher vocational college/bachelor’s degree or above), mother’s education (same categories as father’s), father’s occupation (other/freelancer/farmer/self‐employed business owner/enterprise employee/government or public institution; listed in ascending order of job stability and prestige), mother’s occupation (same categories as father’s), active school club participation (yes/no), and institution type (vocational college/undergraduate college).

#### 3.2.1. Family SES

Previous studies used parental education, occupation, income, household wealth, and property value to construct SES [13, 26–29]. As income, household wealth, and property data were unavailable in this cross‐sectional study, SES was derived from five variables (residence, father’s education, mother’s education, father’s occupation, and mother’s occupation) using principal component analysis (PCA) in SPSS 26.0. Raw data were standardized (*z*‐scores), and components with eigenvalues > 1 were extracted. The standardized score of the first principal component served as the continuous SES measure, with higher values indicating greater socioeconomic advantage [30, 31]. SES validity was assessed via internal coherence (correlations with its five constituent variables) and external validation (correlations with theoretically related variables) [30, 31]. High SES families establish cumulative advantages through economic capital, cultural capital, and social capital. These advantages foster greater involvement in extracurricular activities to enhance comprehensive competitiveness while simultaneously providing material, cognitive, and opportunity‐based support for enrollment in higher‐tier institutions. Conversely, low SES families face relative disadvantages in both domains due to resource constraints and socioeconomic pressures, thus contributing to stratified educational opportunities [26, 32, 33]. Consequently, institution type (vocational college vs. undergraduate college) and active school club participation were selected as external validation indicators of SES in this study.

#### 3.2.2. FRQ

Confirmatory factor analysis (CFA) integrated three key variables (relationship with father, relationship with mother, and parental relationship) into a single latent factor (FRQ) using Mplus 8.3. The model specified FRQ as the latent factor, with the loading for “parental relationship” fixed at 1 for identification (df = 1). Weighted least squares estimation for ordered categorical variables was used. Higher factor scores indicated better FRQ.

#### 3.2.3. GSE

GSE was measured using the 10‐item General Self‐Efficacy Scale (scores 10–40; higher scores indicate greater self‐efficacy). The scale has been widely used and validated in nursing students [34, 35].

### 3.3. Data Analysis

Analyses used SPSS 26.0 and Mplus 8.3. Continuous and categorical variables are presented as mean ± SD and frequencies (percentages), respectively. Analyses proceeded as follows: (1) Constructed SES (PCA) and FRQ (CFA). (2) Examined effects of SES and FRQ on GSE using linear regression. (3) Tested FRQ’s mediating role in the relationship between SES and GSE using PROCESS Macro Model 4, with stratified analysis by institution type. (4) Examined institution type’s moderating effect using PROCESS Macro Model 15.

## 4. Results

### 4.1. PCA

As shown in Table 1, Spearman correlations revealed significant positive associations between SES and all internal coherence variables (all *p* < 0.001). Higher SES correlated with higher parental education, more prestigious/stable parental occupations, and more urban residence. Significant positive correlations were also observed with external validity variables (all *p* < 0.05). Higher SES correlated with students being more likely to study at an undergraduate institution and actively participate in school clubs. To examine whether SES variability differed by institution type, we compared SES between undergraduates and vocational students. The mean SES of undergraduate nursing students and vocational nursing students was 0.060 ± 1.003 and −0.102 ± 0.986, respectively. An independent samples *t*‐test revealed that the SES of undergraduate nursing students was significantly higher than that of vocational nursing students (*t* = 3.017, *p* = 0.003). Importantly, Levene’s test suggested no evidence of unequal variances (*F* = 1.384, *p* = 0.240) and the within‐group SDs were very similar. This indicates that SES dispersion among vocational students was not markedly more homogeneous than among undergraduates, suggesting that restricted SES variability is unlikely to be the only explanation.

**TABLE 1:** Analysis of the association between SES and variables.

	Variable	Spearman correlation coefficient
Internal coherence	Residence	0.529^∗∗∗^
	Father’s education	0.662^∗∗∗^
	Mother’s education	0.712^∗∗∗^
	Father’s occupation	0.718^∗∗∗^
	Mother’s occupation	0.610^∗∗∗^

External validity	Active school club participation	0.062^∗^
	Institution type	0.093^∗∗∗^

^∗^ *p* < 0.05.

^∗∗∗^ *p* < 0.001.

### 4.2. CFA

The model fit indices indicated a mixed but acceptable fit to the data: *χ* ^2^(1) = 70.423, *p* < 0.001; comparative fit index (CFI) = 0.991; Tucker–Lewis index (TLI) = 0.974; root mean square error of approximation (RMSEA) = 0.218 (90% CI: 0.177, 0.263); and standardized root mean square residual (SRMR) = 0.019. We interpreted RMSEA with caution because methodological work shows that RMSEA can falsely indicate poor fit in correctly specified models when the degrees of freedom are very small (df = 1) [36]. Prior studies further suggest that CFI and SRMR are less susceptible to df‐related inflation and can provide more useful information for evaluating very small‐df models [37]. More broadly, fit evaluation is recommended to rely on multiple complementary indices rather than any single index or universal cutoff, and a common approach is to interpret SRMR alongside an incremental fit index such as CFI/TLI [38, 39]. Given the excellent CFI/TLI and very low SRMR, together with strong and statistically significant standardized factor loadings (0.849–0.883, *p* < 0.001), the unidimensional FRQ measurement model was retained. The squared multiple correlations (*R* ^2^) for the observed variables ranged from 0.720 to 0.780, suggesting that the latent factor explained a substantial proportion of variance in each indicator. Overall, the results provide support for the unidimensional structure of FRQ as measured by “relationship with father,” “relationship with mother,” and “parental relationship,” justifying the use of the latent factor scores in further analyses.

### 4.3. Demographic Characteristics

Data from 1461 nursing students were analyzed (Table 2). The mean GSE score was 27.882 ± 4.099; the mean age was 19.580 ± 1.297 years. Participants were predominantly female (82.6%), undergraduates (62.9%), non‐only‐children (79.5%), and active in school clubs (51.2%). Grade distribution was balanced except for fewer fourth‐year students (attributable to vocational programs lacking a fourth year).

**TABLE 2:** Basic characteristics of nursing students.

**Variables**		***n***	**%**

Gender	Male	254	17.4
	Female	1207	82.6

Grade	1	434	29.7
	2	425	29.1
	3	390	26.7
	4	212	14.5

Institution type	Vocational college	542	37.1
	Undergraduate college	919	62.9

Only‐child status	Yes	300	20.5
	No	1161	79.5

Actively participate in school clubs	Yes	748	51.2
	No	713	48.8

Age (mean ± SD)	19.580 ± 1.297		

GSE (mean ± SD)	27.882 ± 4.099		

### 4.4. Multiple Linear Regression

Multiple linear regression analysis, adjusting for covariates (age, gender [dummy‐coded], only‐child status [dummy‐coded], grade [dummy‐coded with first‐year as reference], and institution type [dummy‐coded]), revealed that both FRQ and SES were significantly and positively associated with GSE. This covariate coding scheme was consistently applied in subsequent mediation and moderation analyses. As presented in Table 3, FRQ demonstrated a strong association with GSE (*B* = 1.237, *p* < 0.001), while SES showed a significant yet modest association (*B* = 0.313, *p* = 0.004). This significant positive association between SES and GSE provides support for Hypothesis 1. Furthermore, Pearson correlation analyses indicated significant positive relationships between SES and FRQ (*r* = 0.067, *p* = 0.010), as well as between SES and GSE (*r* = 0.078, *p* = 0.003). A significant positive correlation was also found between FRQ and GSE (*r* = 0.228, *p* < 0.001). Importantly, the SES‐FRQ and SES‐GSE correlations were small in magnitude, indicating limited practical significance and highlighting the value of focusing on the pathway and context‐dependent effects tested in the moderated mediation model.

**TABLE 3:** Multiple linear regression analysis predicting GSE^a^.

	*B*	SE	*β*	*t*	*p*
Constant	24.014	2.678		8.966	< 0.001
FRQ	1.237	0.144	0.220	8.600	< 0.001
SES	0.313	0.109	0.076	2.872	0.004

^a^Adjusted for age, gender, only‐child status, grade, institution type.

### 4.5. Mediation Analysis

Stratified mediation analyses using PROCESS Model 4 revealed significant institutional differences in the pathway from SES to GSE through FRQ, adjusting for age, gender, only‐child status, and grade (institution type additionally controlled for total sample). As detailed in Table 4, the total sample exhibited partial mediation wherein SES exerted significant total (*β* = 0.373, 95% CI [0.154, 0.592]) and direct (*β* = 0.313, 95% CI [0.099, 0.527]) effects on GSE, with FRQ mediating 16.1% of this association (indirect effect: *β* = 0.060, 95% CI [0.009, 0.115]). The significant indirect effect of SES on GSE through FRQ in the total sample supports Hypothesis 2, which proposed the mediating role of FRQ. However, stratified analyses revealed divergent patterns by institution type. Vocational students demonstrated nonsignificant total (*β* = −0.019, 95% CI [−0.379, 0.342]), direct (*β* = −0.014, 95% CI [−0.372, 0.344]), and indirect effects (*β* = −0.005, 95% CI [−0.060, 0.051]), indicating an absence of mediation despite FRQ independently predicting GSE (*β* = 0.695, *t* = 2.878, *p* < 0.01). Conversely, undergraduates showed robust partial mediation with significant total (*β* = 0.618, 95% CI [0.344, 0.892]), direct (*β* = 0.500, 95% CI [0.234, 0.765]), and indirect effects (*β* = 0.118, 95% CI [0.043, 0.201]), the latter accounting for 19.1% of SES’s influence and supported by significant SES ⟶ FRQ (*β* = 0.078, *t* = 3.130, *p* < 0.01) and FRQ ⟶ GSE (*β* = 1.509, *t* = 8.490, *p* < 0.001) pathways.

**TABLE 4:** The mediating effect of SES on GSE via FRQ across different samples.

	**Total samples^a^ *n* = 1461**				**Vocational samples^b^ *n* = 542**				**Undergraduate samples^b^ *n* = 919**			
	**FRQ**		**GSE**		**FRQ**		**GSE**		**FRQ**		**GSE**	
	***β***	***t***	***β***	***t***	***β***	***t***	***β***	***t***	***β***	***t***	***β***	***t***
SES	0.048	2.438^∗^	0.313	2.872^∗∗^	−0.007	−0.214	−0.014	−0.077	0.078	3.130^∗∗^	0.500	3.697^∗∗∗^
FRQ			1.237	8.600^∗∗∗^			0.695	2.878^∗∗^			1.509	8.490^∗∗∗^
*R* ^2^	0.016		0.062		0.030		0.034		0.016		0.106	
*F*	2.918^∗∗^		10.569^∗∗∗^		2.792^∗^		2.699^∗∗^		2.123^∗^		13.558^∗∗∗^	
Total effect (95% CI)	0.373 (0.154, 0.592)				−0.019 (−0.379, 0.342)				0.618 (0.344, 0.892)			
Direct effect (95% CI)	0.313 (0.099, 0.527)				−0.014 (−0.372, 0.344)				0.500 (0.234, 0.765)			
Indirect effect (95% CI)	0.060 (0.009, 0.115)				−0.005 (−0.060, 0.051)				0.118 (0.043, 0.201)			

^a^Adjusted for age, gender, only‐child status, grade, institution type.

^b^Adjusted for age, gender, only‐child status, grade.

^∗^ *p* < 0.05.

^∗∗^ *p* < 0.01.

^∗∗∗^ *p* < 0.001.

### 4.6. Moderated Mediation Analysis

**FIGURE 1:**
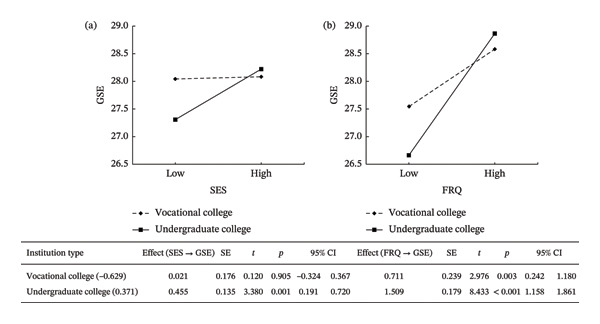
Conditional effects of SES and FRQ on GSE moderated by institution type.

The moderated mediation model (PROCESS Model 15) examined the association between SES and GSE through the mediator (FRQ), with institution type as a moderator. Covariates included grade, age, gender, and only‐child status. As shown in Table 5, SES was positively associated with FRQ (*β* = 0.047, *t* = 2.394, *p* < 0.05) and with GSE (*β* = 0.294, *t* = 2.702, *p* < 0.01). FRQ was positively associated with GSE (*β* = 1.213, *t* = 8.449, *p* < 0.001). The interaction between SES and institution type was significant (*β* = 0.434, *t* = 1.993, *p* < 0.05), suggesting that the relationship between SES and GSE varies depending on the institution type. Specifically, SES has a stronger positive effect on GSE in undergraduate programs. Similarly, the interaction between FRQ and institution type was significant (*β* = 0.798, *t* = 2.679, *p* < 0.01), indicating that the impact of FRQ on GSE is also stronger aiming undergraduate students. This result shows that family resources are more beneficial in an academic setting that demands higher cognitive and emotional investment, as seen in undergraduate programs compared to vocational ones. The significant interaction between SES and institution type, with a stronger direct effect observed among undergraduate students, confirms Hypothesis 3. Furthermore, simple slope analysis (Figure 1) displayed that SES positively associated with GSE among undergraduates (*β*_simple_ = 0.455, *p* = 0.001) but not among vocational students (*β*_simple_ = 0.021, *p* = 0.905). Interestingly, FRQ was positively associated with GSE in both undergraduates (*β*_simple_ = 1.509, *p* < 0.001) and vocational students (*β*_simple_ = 0.239, *p* = 0.003), and the association was larger among undergraduates. In addition, the conditional indirect effect of SES on GSE through FRQ differed significantly by institution type: significant for undergraduates (*β* = 0.072, 95% CI [0.012, 0.139]) and vocational students (*β* = 0.034, 95% CI [0.002, 0.080]), though smaller in magnitude for the latter. This difference was confirmed by a significant index of moderated mediation (index = 0.038, 95% CI [0.001, 0.094]), demonstrating that institution type moderated the indirect effect of SES on GSE via FRQ. This significant index of moderated mediation demonstrates that the strength of the indirect effect depends on institution type, thereby validating Hypothesis 4.

**TABLE 5:** The moderating effect of institution type on the mediation process^b^.

	**FRQ**		**GSE**	
	***β***	***t***	***β***	***t***
SES	0.047	2.394^∗^	0.294	2.702^∗∗^
Institution type			−0.302	−1.302
SES × institution type			0.434	1.993^∗^
FRQ			1.213	8.449^∗∗∗^
FRQ × institution type			0.798	2.679^∗∗^
*R* ^2^	0.015		0.069	
*F*	3.256^∗∗^		9.760^∗∗∗^	

^b^Adjusted for age, gender, only‐child status, grade.

^∗^ *p* < 0.05.

^∗∗^ *p* < 0.01.

^∗∗∗^ *p* < 0.001.

## 5. Discussion

Guided by COR theory, this study established and tested a moderated mediation model to investigate the pathways between SES and GSE in nursing students, with FRQ as the mediator and institution type as the moderator. The results revealed three key findings: (1) SES was positively associated with GSE; (2) FRQ partially mediated the relationship between SES and GSE; and (3) institution type moderated both the direct association of SES with GSE and the indirect association of FRQ with GSE, with stronger effects observed among undergraduate students. Collectively, these findings offer theoretical insights into the interplay between structural family resources, family relational functioning, and educational contexts in shaping GSE of nursing students.

The direct positive association between SES and GSE aligns with COR theory, supporting the premise that resource advantage is linked to greater confidence in overcoming challenges. High SES families provide abundant educational resources, social capital, and economic stability [13], which collectively enhance individuals’ confidence in their ability to overcome challenges [7, 40]. Our findings are consistent with previous studies, indicating that socioeconomic advantages contribute to greater self‐efficacy through resource accumulation and reduced vulnerability to resource loss [26, 32]. Notably, the direct effect of SES on GSE was modest (*B* = 0.313), suggesting that SES is one of the multiple factors influencing GSE. Additionally, the bivariate correlations between SES and FRQ/GSE were statistically significant but very small, suggesting limited practical importance in isolation; therefore, the conditional process model provides a more meaningful account of the context‐dependent mechanism. This aligns with studies showing that individual characteristics (e.g., resilience) and environmental factors (e.g., peer support) also play important roles [8, 10]. Nevertheless, the significance of SES persists, highlighting the need to address resource disparities to promote psychological development among nursing students.

The mediation results suggested that FRQ is one relational pathway linking SES to GSE, particularly in the total sample and the undergraduate subgroup. This finding supports the theoretical framework that family environment operates through both structural/material (SES) and relational (FRQ) dimensions to predict self‐efficacy [13, 14]. Importantly, these dimensions are not independent; higher SES often facilitates a more harmonious and supportive family environment (e.g., higher FRQ) by reducing economic pressure and parental stress [13, 18, 41], which in turn further enhances self‐efficacy. Consistent with prior evidence, SES is positively associated with FRQ, suggesting that high SES reduces economic stress, alleviates parental psychological distress, and improves marital and parent–child relationships [18, 19]. In turn, FRQ is significantly linked to GSE, indicating that a supportive family environment characterized by low conflict, high cohesion, and frequent emotional sharing provides individuals with stable sources of confidence and successful coping experiences [23]. This emphasis on self‐efficacy is further supported by recent evidence linking higher self‐efficacy with stronger professional identity among nursing students, suggesting that enhancing GSE may carry downstream benefits for broader professional development outcomes [42]. Research has confirmed that positive family relationships serve as a critical buffer, fostering the belief in one’s ability to overcome challenges [8, 13]. This is particularly beneficial for nursing students who face intense academic and clinical pressures. In summary, FRQ acts as a pathway that facilitates the conversion of structural family resources (SES) into psychosocial resources. Higher family SES likely reduces economic strain and parental distress, which fosters a supportive and harmonious family environment, thereby enhancing FRQ. This positive family environment, in turn, significantly contributes to greater GSE, as it provides individuals with emotional stability and coping resources.

The moderated mediation analysis revealed that institution type (vocational college vs. undergraduate college) significantly moderates both the direct and indirect pathways, with stronger effects observed among undergraduate students. This discrepancy can be explained by differences in educational models and student experiences across institutions. Vocational colleges emphasize hands‐on training and tailored instruction, which may enhance self‐efficacy through practical problem‐solving opportunities [7]. This focus on applied skills could mitigate the influence of SES and FRQ, as students’ confidence is more closely tied to clinical competence acquired through training rather than family resources or emotional support. For example, simulation‐based educational designs embedded in nursing training have been shown to yield measurable gains in learners’ self‐confidence and related learning outcomes, illustrating how institution‐provided practice infrastructures can directly build psychological capital [43]. From a COR perspective, vocational programs may provide comparatively strong institutional resources (e.g., structured supervision, skills laboratories, repeated practice opportunities, and frequent performance feedback) that function as an alternative resource reservoir. Such institutional resources may substitute for family‐derived resources, thereby weakening the extent to which students’ GSE depends on SES or FRQ. In addition, repeated mastery‐oriented training may represent resource investment that initiates a gain spiral: early gains in clinical competence (a valued resource) may build self‐efficacy (a psychological resource), which may further motivate engagement and persistence, leading to additional competence gains and reduced vulnerability to resource loss under training demands. Conversely, undergraduate nursing programs involve heavier academic burdens and higher clinical expectations, which may increase the salience of family‐derived resources for sustaining psychological capital during training [9]. In this context, high SES provides critical resources (e.g., access to advanced learning materials, mentorship) to cope with academic stress, while positive FRQ offers emotional resilience, making both factors more impactful for GSE. Additionally, undergraduate students may have more diversified demands for their future careers, potentially making them more dependent on family resources and support in their career planning. This further amplifies the impact of SES and FRQ on GSE. Recent evidence indicates that nursing students’ stress profiles and coping needs vary across educational stages, with academic stress showing significant differences by educational level, alongside variation in clinical workload and practice settings demands [44, 45]. Building on this broader training and curriculum demand explanation, recent mixed‐method evidence suggests that how nursing programs allocate and integrate specialized content can shape students’ perceived confidence/self‐efficacy, underscoring that curriculum structure itself is a plausible institutional driver of psychological resources [46]. This is consistent with COR theory’ s emphasis on resource loss threat under high demand [47]. Additionally, unmeasured school‐specific characteristics could contribute to differences attributed to institution type [48, 49]. These findings necessitate the integration of institutional characteristics into the design of any interventions aimed at bolstering nursing students’ GSE.

These results extend the COR theory by illustrating how family systems function as resource reservoirs that influence self‐efficacy through both material and emotional channels. They also emphasize the role of educational contexts in modulating these relationships. From a COR perspective, interventions to support nursing students’ GSE should aim to reduce resource loss threat in high‐demand training contexts and build resource gain processes that accumulate psychological capital over time. More precisely, for undergraduate colleges, where academic and clinical demands may heighten resource loss threat, it is important to prioritize support for students from lower SES backgrounds by providing readily accessible academic resources (e.g., tutoring, learning support, and academic communication coaching) and by strengthening relational resources through family engagement initiatives to improve FRQ; together, these supports may help protect students’ psychological capital and facilitate resource gain. For vocational colleges, leveraging strengths in practice‐based education can be framed as promoting resource investment and gain spirals. Embedding mastery‐oriented skill‐building modules (e.g., simulation‐based practice, stepwise competency milestones, and structured feedback) may help students accumulate competence resources that translate into self‐efficacy gains, particularly for those with fewer family resources. Across institution, integrating family relationship education into nursing curricula (e.g., workshops on communication skills and conflict management) can be positioned as building relational resources that contribute to students’ broader resource caravans, thereby supporting GSE regardless of institution type.

## 6. Limitations and Future Research

Several limitations should be acknowledged. First, the cross‐sectional design precludes causal inferences. Longitudinal or experimental studies are needed to validate the causal pathways proposed. Second, self‐reported measures may introduce social desirability bias. Third, our SES measure did not include direct economic indicators (e.g., household income and assets). In addition, the SES distribution was similar across institution types, which may have reduced statistical power to detect SES‐related associations, particularly in subgroup analyses. Future studies should incorporate objective economic indicators to better capture SES variability. Fourth, the sample was drawn from only two institutions in a single region, which may limit external validity and raises the possibility that school‐specific characteristics (e.g., campus culture, student composition, and support services) may partially account for differences attributed to institution type. Multicenter studies involving diverse cultural and educational contexts are recommended. Finally, other potential mediators (e.g., social support) and moderators (e.g., personality traits and coping styles) were not examined and warrant further investigation.

## 7. Conclusion

Our findings elucidate a dual‐pathway mechanism: SES was positively associated with nursing students’ GSE through both a direct pathway and an indirect pathway mediated by FRQ, with the magnitude of these effects being more substantial in undergraduate programs compared to vocational institutions. These findings highlight the importance and relevance of structural family resources and family relational functioning for nursing students’ GSE, while underscoring that educational context may shape the strength of these associations. Nursing education and management initiatives may therefore benefit from context‐sensitive student support strategies that combine academic/skills support with attention to family and relational resources.

## Funding

This work was supported by the Science and Technology of Wuhu City, Grant No. 2023yf103; the Department of Education of Anhui Province, Grant No. 2024cxtd236; the Anhui College of Traditional Chinese Medicine, Grant No. 2024xjjxyj10; and Wannan Medical College, Grant No. WK2024ZQNZ16.

## Conflicts of Interest

The authors declare no conflicts of interest.

## Data Availability

The data that support the findings of this study are available from the corresponding author upon reasonable request.
